# Association of device-measured physical activity and sedentary behaviour with cardiovascular risk factors, health-related quality-of-life and exercise capacity over 12-months in cardiac rehabilitation attendees with coronary heart disease

**DOI:** 10.1186/s13102-022-00562-7

**Published:** 2022-09-07

**Authors:** Nicole Freene, Margaret McManus, Tarryn Mair, Ren Tan, Rachel Davey

**Affiliations:** 1 Physiotherapy, Faculty of Health, University of Canberra, Bruce, ACT Australia; 2 Health Research Institute, University of Canberra, Bruce, ACT Australia; 3 Canberra Health Services, Garran, ACT Australia; 4 Exercise Physiology, Canberra Health Services, Garran, ACT Australia

**Keywords:** Physical activity, Sedentary behaviour, Cardiovascular risk, Cardiac rehabilitation, Accelerometer

## Abstract

**Background:**

Few studies have considered the relationship between risk factors, physical activity and sedentary behaviour in people with heart disease. Here we examine the independent relationship of device-measured physical activity and sedentary behaviour on risk factors, quality-of-life and exercise capacity over 12-months in cardiac rehabilitation attendees.

**Methods:**

Hospital-based phase II cardiac rehabilitation participants with coronary heart disease were assessed at the start and end of cardiac rehabilitation (6-weeks), 6 and 12-months. Physical activity (moderate-to-vigorous (MVPA), light-intensity (LIPA); min/day) and sedentary behaviour (min/day, bouts, breaks) were measured using an ActiGraph accelerometer. Risk factors included waist circumference, body mass index, systolic blood pressure (SBP), fasting blood lipid and glucose levels, anxiety and depression. Quality-of-life and exercise capacity were also collected. Associations were assessed with Generalized Estimating Equation modeling.

**Results:**

Sixty-seven participants were included (mean age = 64 (SD 9) years; 81% male). An association was found between higher MVPA and lower high density lipoprotein (*p* ≤ 0.001). No significant (*p* ≤ 0.001) associations were found between sedentary behaviour variables and other outcomes. At *p* < 0.05 several associations were significant. Increased MVPA and LIPA were associated with decreased total cholesterol. Higher MVPA was associated with decreased SBP, whereas higher LIPA was associated with decreased waist circumference and body mass index. Higher sedentary behaviour bouts and breaks were associated with increased total cholesterol, anxiety and depression, and decreased SBP over time.

**Conclusions:**

Any intensity of physical activity was associated with decreased total cholesterol. Increased LIPA was associated with improved measures of adiposity, while breaking up sedentary behaviour and increasing MVPA may decrease SBP over time. Further investigation of MVPA, LIPA and the distribution of sedentary behaviour is indicated in cardiac rehabilitation attendees to explore their relationship with risk factors.

*Trial registration*: Australian New Zealand Clinical Trials Registry (ANZCTR): ACTRN12615000995572, http://www.ANZCTR.org.au/ACTRN12615000995572.aspx. Registered 22 September 2015.

**Supplementary Information:**

The online version contains supplementary material available at 10.1186/s13102-022-00562-7.

## Background

Nearly 200 million people worldwide were diagnosed with coronary heart disease (CHD) in 2019 [1]. Myocardial infarctions are a common manifestation of CHD, with approximately one in three being repeat events [2]. Not only are repeat cardiac events more likely to be fatal, they cost Australia, the country of this study, more than $8.4 billion annually [2]. In people with CHD, physical inactivity is an independent risk factor for all-causes of death [3]. Consequently, within cardiac rehabilitation programs, an integral component of recovery from cardiac events, participants internationally are encouraged to meet the public health physical activity guidelines to improve health outcomes, that is, achieve at least 150-min of moderate-to-vigorous intensity physical activity (MVPA) per week [4–6]. However, the relationship between physical activity, sedentary behaviour and risk factors for recurrent cardiac events [4–6], such as blood pressure, weight, blood glucose and lipid levels, anxiety and depression within cardiac rehabilitation is unclear.

There have been conflicting findings from systematic reviews and meta-analyses regarding the effects of exercise-based cardiac rehabilitation on all-cause and cardiovascular mortality and hospital admissions. A Cochrane review published in 2016 found that exercise-based cardiac rehabilitation reduced the risk of cardiovascular mortality and improved health-related quality-of-life, with a reduction in hospital admissions in the short-term, compared with no-exercise control [7]. There was no effect on the risk of repeat myocardial infarctions and revascularisation procedures. In a contemporary review of the literature (studies published year 2000+), Powell et al. found no effect on all-cause and cardiovascular mortality when exercise-based cardiac rehabilitation was compared to no-exercise control, with a small reduction in hospital admissions [8]. Neither of these reviews reported on the effects of exercise-based cardiac rehabilitation on risk factors, however, other reviews have reported improvements in risk factors [9]. In addition, neither of the reviews considered the physical activity or sedentary behaviour levels of cardiac rehabilitation attendees. Exercise is defined as a subset of physical activity, and at present there is only moderate evidence that physical activity increases with cardiac rehabilitation participation [10].

Few single studies have investigated the association of physical activity and sedentary behaviour with risk factors in people with CHD that have attended cardiac rehabilitation. Studies that have measured physical activity (MVPA, steps) and sedentary behaviour, either self-reported [11–13] or device-measured [14–16], have found that higher physical activity levels are associated with decreases in waist circumference [14], body mass index (BMI) [14, 15], blood glucose and triglyceride levels [14, 15], depression and anxiety [12]; and increases in high density lipoprotein (HDL) [14, 15] and health-related quality-of-life [11]. In terms of sedentary behaviour, higher levels are associated with decreased exercise capacity [16] and HDL [16], and increased triglycerides [16], BMI [16], waist circumference [16] and anxiety [13]. Most of these studies were cross-sectional and conducted at varying times within and post-cardiac rehabilitation. In a recent systematic review investigating physical activity and sedentary behaviour in the secondary prevention of CHD, increased physical activity resulted in increased 6-min walk test distance (6MWTD), improved quality-of-life and improved blood glucose and lipid levels [17]. However, most studies within the review included structured exercise training, with few studies including physical activity beyond this. According to the US 2018 physical activity and sedentary behaviour guidelines scientific report, in older adults with cardiovascular disease there is limited evidence that increased physical activity increases physical function eg: 6MWTD [18]. Therefore, the evidence for physical activity and sedentary behaviour to manage risk factors, health-related quality-of-life and exercise capacity in cardiac rehabilitation attendees with CHD is currently limited, and no studies have investigated both physical activity and sedentary behaviour associations with risk factors over time. Thus, the research questions for our study were:

1. What is the independent relationship of device-measured physical activity on the cardiovascular risk factors, health-related quality-of-life and exercise capacity of people over 12-months after starting cardiac rehabilitation?
2. What is the independent relationship of device-measured sedentary behaviour on cardiovascular risk factors, health-related quality-of-life and exercise capacity of people over 12-months after starting cardiac rehabilitation?

## Methods

### Design

Using a prospective cohort study design, 72 participants aged ≥ 18 years and enrolled in the 6-week Australian hospital-based phase II cardiac rehabilitation program were recruited to the 12-month observational study between November 2015 and August 2016. The cardiac rehabilitation program is multidisciplinary, time-limited (12 sessions [2 per week for 6 weeks]), conducted in groups, and has educational and supervised exercise components (one hour education plus one hour exercise). Participants were included if they had stable CHD and were receiving optimal medical treatment ± revascularisation [19]. All participants provided written consent. The study protocol, baseline, 6-week and 6 and 12-month results have been described elsewhere [19–21]. The investigation conforms with the principles outlined in the Declaration of Helsinki [22]. Reporting was guided by the STROBE Statement (cohort studies) (Additional file 1).

### Exposures

A triaxial commercial accelerometer (ActiGraph ActiSleep, Fort Walton Beach, FL) was used to objectively assess sedentary behaviour (min/day, bouts, breaks) and physical activity (MVPA, light-intensity (LIPA); min/day). Participants were asked to wear the monitor on their right hip for 24 h/day for 7-consecutive days by cardiac rehabilitation staff. Sleep time was eliminated by using a time filter applied from 0700 to 2230 h, the average wake and sleep time reported by participants. All data was sampled and downloaded as raw data (30 Hz), converted to 1-s epochs (time interval), and then counts per minute (cpm) using the Actilife software. Data was screened, excluding data with < 10 h/day wear time (non-wear defined as > 60 consecutive minutes of zero activity, allowing for 2 min of counts between 0 and 100) and < 4 days of valid data. The Freedson Combination energy expenditure algorithm was used to determine time spent in LIPA (100–1951 cpm), MVPA (≥ 1952 cpm) and sedentary behaviour (< 100 cpm) [23]. Estimating time spent in physical activity and sedentary behaviour was calculated by dividing the total time spent (minutes) in each threshold by the number of valid days. Sedentary behaviour bout data used a minimum length of 10 min, with no drop time [16]. Sedentary bouts are the number of bouts (≥ 10 consecutive minutes) of sedentary time per day. Average sedentary bout length is the total time in sedentary bouts divided by the total number of bouts per day. A break is an interruption in sedentary time (≥ 100 cpm).

### Outcome measures

Outcomes included BMI (kg/m2); waist circumference; resting systolic blood pressure; fasting blood lipid (total cholesterol, HDL) and glucose levels; exercise capacity (6MWTD [24]); health-related quality of life (MacNew Heart Disease Health-related Quality of Life Questionnaire Global score (MacNew Global), with scores from 1, low health-related quality of life, to 7, high health-related quality of life [25]); and anxiety and depression (Hospital Anxiety and Depression Scale total score (HADS total), with maximal score of 42, high scores indicating high anxiety and depression [26]). The MacNew and HADS have good reliability and validity in adults with cardiovascular disease. Sociodemographic data was collected. All exposures and outcome measures (physical activity, sedentary behaviour, cardiovascular risk factors, health-related quality-of-life and exercise capacity) were assessed at baseline, 6-weeks, and 6 and 12-months.

### Data analysis

Data were analyzed using SPSS version 27. Descriptive statistics were reported using means and SDs, medians with IQRs or proportions, where appropriate. Normality was assessed using the Shapiro–Wilk test. Accelerometer missing data was considered and differences in baseline characteristics (age, gender, education, employment, 6MWTD) between participants with missing data and participants without missing data were assessed using independent t-tests or Mann–Whitney U tests, where appropriate. Missing data was handled by bringing the last value forward.

Models using the Generalized Estimating Equations (GEE) approach were used to investigate the association between physical activity and sedentary behaviour and cardiovascular risk factors, health-related quality-of-life and exercise capacity over 12-months after starting cardiac rehabilitation. Physical activity and sedentary behaviour measures (LIPA min/day, MVPA min/day, sedentary behaviour min/day, sedentary behaviour bouts/day, sedentary behaviour breaks/day, average sedentary behaviour bout length min/day: *independent variables*) were modelled separately with cardiovascular risk factors (waist circumference, BMI, systolic blood pressure, blood lipid and glucose levels, anxiety and depression (HADS total)), health-related quality of life (MacNew Global) and 6MWTD as response outcomes (*dependent variables*) over the 4-time points. Interaction terms involving Time (moderator) and each independent variable were added in the models to assess if the effect predictor was significantly changing over time. The effects of independent variables were reported as regression coefficients with their associated 95% CIs. To account for multiple tests, a Bonferroni correction was applied (6 × 9 = 54) with *p* ≤ 0.001 used as the threshold for statistical significance. All models were adjusted for total accelerometer counts/day, age, gender, education and employment. Age, gender, education and employment are known factors to be associated with physical activity and sedentary behaviour levels [27]. In addition, systolic blood pressure models were also adjusted for blood pressure medications, total cholesterol and HDL models were adjusted for cholesterol medications, and blood glucose level models were adjusted for type 2 diabetes. Total accelerometer counts/day is a proxy for total physical activity volume, encompassing frequency, intensity and duration of activity bouts [28, 29], although it is understood that it may be highly correlated with MVPA, LIPA and sedentary behaviour. Thus, sensitivity analyses were performed where GEE models were not adjusted for total accelerometer counts/day to determine if this changed findings. Additional sensitivity analyses were also performed exploring on-protocol and unadjusted models.

## Results

**Fig. 1:**
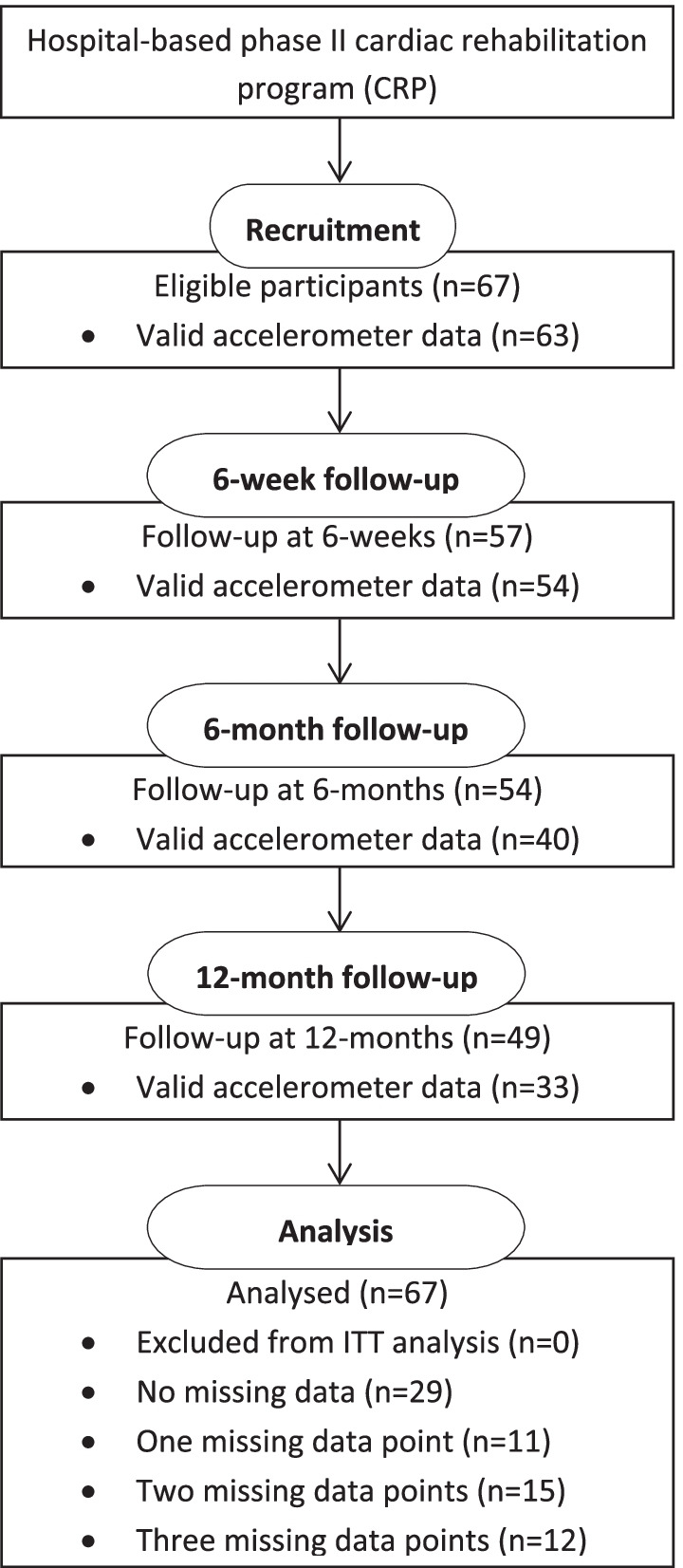
Flow chart of participants with accelerometer data through the 12-month trial

Sixty-seven participants provided accelerometer data throughout the 12-month period (Fig. 1). Accelerometers were worn for a median of 7 (IQR 7, 8) days, with a median wear time per day of 14 (IQR 13.5, 14.7) hours. Participant characteristics at baseline are shown in Table 1. Most participants had undergone a percutaneous coronary intervention, were male, tertiary educated and approximately half were working in paid employment. On average, participants were 64 years old; obese; with normal blood pressure, lipid and glucose levels; reporting low levels of anxiety and depression; and high health-related quality of life. Adherence to the cardiac rehabilitation program was high with 90% of participants (n = 60/67) completing all 12 sessions. Participants with missing accelerometer data (n = 38, 68% (n = 26/38) with 2–3 accelerometer measures) and those without missing accelerometer data (n = 29) did not differ at baseline for age, gender, education, employment and 6MWTD.

**Table 1:** Characteristics of participants at baseline

Characteristic	(n = 67)
Age (year), mean (SD)	64.2 (9.4)
Gender, number male (%)	54 (81)
Diagnosis, number percutaneous coronary intervention (%)	53 (79)
Education, number tertiary educated (%)	39 (71)
Employment, number paid employment (%)	29 (52)
Blood pressure medication, number yes (%)	47 (86)
Cholesterol medication, number yes (%)	49 (89)
Type 2 diabetes, number yes (%)	13 (23)
*Measures of disease risk and fitness*	
Waist circumference (cm), median (IQR)	103 (95.5, 113.3)
BMI (kg/m^2^), median (IQR)	29.7 (26.2, 32.6)
Systolic blood pressure (mmHg), mean (SD)	125 (12.8)
MacNew Global (1–7), median (IQR)	5.89 (5.07, 6.17)
HADS-total (1–42), median (IQR)	5 (3, 9)
Total cholesterol (mmol/L), median (IQR)	3.5 (2.9, 4)
High-density lipoprotein (mmol/L), median (IQR)	1.05 (0.9, 1.1)
Blood glucose level (mmol/L), median (IQR)	5.6 (5, 6.35)
6-min walk test distance (m), median (IQR)	480 (435, 528)
*Physical activity and sedentary behaviour variables*^*a*^	
MVPA (min/day), median (IQR)	32.7 (24.6, 49.9)
LIPA (min/day), median (IQR)	71.5 (57.1, 84.4)
SB (min/day), mean (SD)	723.3 (58.4)
Number SB bouts/day, mean (SD)	13.1 (4)
Duration SB bouts/day (min), mean (SD)	19.4 (2.3)
Number SB breaks/day, mean (SD)	13 (4)

*HADS* hospital anxiety and depression scale, *MVPA* moderate-to-vigorous physical activity, *LIPA* light-intensity physical activity, *SB* sedentary behaviour

^a^ActiGraph accelerometer 1-s epochs

### Association between device-measured physical activity and cardiovascular risk factors, health related quality of life and exercise capacity

A significant association was found between MVPA and HDL (*p* < 0.001). Regardless of time, HDL decreased by 0.002 mmol/L for each min/day increase in MVPA. Although not perceptible, the change in the effect of MVPA on HDL over time was also significant (*p* < 0.05). Table 2 shows the results of the GEE models for physical activity variables. Regardless of the amount of LIPA, 6MWTD significantly increased over time (*p* ≤ 0.001). Higher levels of MVPA were also associated with increased waist circumference and BMI, and decreased systolic blood pressure, total cholesterol and 6MWTD, regardless of time. Over time, total cholesterol increased by 0.001 mmol/L for each min/day increase in MVPA. Higher levels of LIPA were associated with decreased waist circumference, BMI and total cholesterol, regardless of time. These relationships did not reach the threshold for statistical significance but were significant at the level of *p* < 0.05. No physical activity variables were significantly associated with health-related quality-of-life, anxiety and depression and blood glucose levels.

**Table 2:** Associations between physical activity variables and cardiovascular risk factors, health-related quality-of-life and exercise capacity over 12-months in cardiac rehabilitation attendees

Independent variable^a^	Dependent variable	Time			Independent variable			Time*Independent variable		
		β	95% CI	*p* value	β	95% CI	*p* value	β	95% CI	*p* value
Moderate-to-Vigorous	Waist circumference (cm)	**0.16**	**0.01, 0.31**	**0.04**	**0.05**	**0.01, 0.1**	**0.03**	−0.01	−0.1, 9.76E−5	0.06
Physical Activity (min/day)	Body mass index (kg/m^2^)	**0.06**	**0.02, 0.11**	**0.01**	**0.01**	**0.004, 0.02**	**0.004**	−0.001	−0.002, 0	0.09
	Systolic blood pressure (mmHg)^b^	−0.03	−0.54, 0.47	0.9	−**0.12**	−**0.20, **−**0.04**	**0.005**	0.001	−0.01, 0.01	0.86
	MacNew Global	0.01	−0.01, 0.03	0.25	−0.001	−0.01, 0.004	0.84	0	−7E−5, 0.001	0.09
	HADS−total	−0.01	−0.13, 0.12	0.93	0.01	−0.02, 0.04	0.46	0	−0.004, 0.003	0.83
	Total cholesterol (mmol/L)^c^	0.005	−0.02, 0.02	0.65	−**0.01**	−**0.01, **−**0.002**	**0.01**	**0.001**	**0, 0.002**	**0.002**
	High−density lipoprotein (mmol/L)^c^	−0.001	−0.01, 0.004	0.74	−**0.002**	−**0.003, **−**0.001**	** < 0.001**	**0**	**2.8E**−**5, 0.001**	**0.03**
	Blood glucose level (mmol/L)^d^	0.04	−0.03, 0.1	0.25	0.003	−0.01, 0.01	0.46	−6.7E−5	−0.001, 0.001	0.90
	6-min walk test distance (m)	1.36	−0.43, 3.15	0.14	−**0.64**	−**1.07, **−**0.20**	**0.004**	0.05	−0.03, 0.12	0.22
Light-intensity	Waist circumference (cm)	0.17	−0.22, 0.56	0.40	−**0.05**	−**0.09, **−**0.002**	**0.04**	−0.002	−0.01, 0.002	0.32
Physical Activity (min/day)	Body mass index (kg/m^2^)	0.08	−0.07, 0.22	0.31	−**0.01**	−**0.03,** −**0.001**	**0.04**	−0.001	−0.002, 0.001	0.52
	Systolic blood pressure (mmHg)^b^	−0.32	−1.17, 0.54	0.47	−0.005	−0.09, 0.08	0.91	0.007	−0.002, 0.02	0.14
	MacNew Global	0.04	−0.01, 0.09	0.08	0.003	−0.003, 0.01	0.31	0	−0.001, 0	0.55
	HADS-total	−0.01	−0.29, 0.27	0.96	0.03	−0.003, 0.06	0.08	0	−0.004, 0.003	0.80
	Total cholesterol (mmol/L)^c^	**0.07**	**0.01, 0.13**	**0.03**	−**0.01**	−**0.01, 0**	**0.04**	0	−0.001, 0	0.14
	High-density lipoprotein (mmol/L)^c^	0.01	−0.001, 0.02	0.07	7.2E−5	−0.002, 0.002	0.93	−4.7E−5	0, 9.6E−5	0.52
	Blood glucose level (mmol/L)^d^	0.1	−0.01, 0.20	0.08	0.001	−0.006, 0.01	0.78	−0.001	−0.002, 0	0.18
	6-min walk test distance (m)	**6.79**	**2.63, 10.95**	**0.001**	0.12	−0.63, 0.86	0.76	−0.04	−0.08, 0.01	0.09

^a^All models adjusted for total counts/day, age, gender, education and employment. Significant results are highlighted in bold. HADS, hospital anxiety and depression scale

^b^Model also adjusted for blood pressure medications

^c^Models also adjusted for cholesterol medications

^d^Models also adjusted for type 2 diabetes

### Association between device-measured sedentary behaviour and cardiovascular risk factors, health related quality of life and exercise capacity

No significant (*p* ≤ 0.001) associations were found between sedentary behaviour variables and cardiovascular risk factors, health-related quality-of-life and exercise capacity. However, at the *p* < 0.05 threshold, several associations were significant. The GEE models for sedentary behaviour variables are shown in Table 3. Regardless of time, higher sedentary behaviour (min/day) levels were negatively associated with 6MWTD but the change in 6MWTD over time is significantly increased with increased sedentary behaviour (min/day). A higher number of sedentary behaviour bouts and breaks were associated with an increase in total cholesterol, anxiety and depression, regardless of time. The change in systolic blood pressure overtime is significantly decreased by an increased number of sedentary behaviour bouts and breaks. The average duration of sedentary behaviour bouts (mins) was not significantly associated with any dependent variables. No sedentary behaviour variables were significantly associated with waist circumference, BMI, health-related quality-of-life, HDL and blood glucose levels. Additionally, health-related quality-of-life and blood glucose levels were not significantly associated with any physical activity or sedentary behaviour variables.

**Table 3:** Associations between Sedentary Behaviour variables and cardiovascular risk factors, health-related quality-of-life and exercise capacity over 12-months in cardiac rehabilitation attendees

Independent variable^a^	Dependent variable	Time			Independent variable			Time*Independent variable		
		β	95% CI	*p*-value	β	95% CI	*p*-value	β	95% CI	*p*-value
Sedentary Behaviour	Waist circumference (cm)	−0.31	0.51, −1.32	0.54	−0.01	−0.02, 0.01	0.29	0	−0.001, 0.002	0.61
(min/day)	Body mass index (kg/m^2^)	0.04	−0.22, 0.29	0.77	−0.001	−0.004, 0.002	0.61	−3.38E−5	0, 0	0.86
	Systolic blood pressure (mmHg)^b^	1.5	−1.63, 4.7	0.34	−0.01	−0.05, 0.03	0.63	−0.002	−0.01, 0.003	0.38
	MacNew Global	0.04	−0.11, 0.18	0.62	0.001	−0.002, 0.004	0.37	−9.55E−6	0, 0	0.93
	HADS-total	−0.58	−1.33, 0.18	0.14	−0.001	−0.01, 0.01	0.78	0.001	0, 0.002	0.14
	Total cholesterol (mmol/L)^c^	−0.10	−0.24, 0.06	0.22	−0.001	−0.002, 0.001	0.34	0	−5.3E−5, 0	0.13
	High-density lipoprotein (mmol/L)^c^	−0.01	−0.05, 0.03	0.66	0	−0.001, 0	0.15	2.3E−5	−3.5E−5, 8.0E−5	0.44
	Blood glucose level (mmol/L)^d^	−0.26	−0.75, 0.24	0.31	−0.001	−0.004, 0.003	0.70	0	0, 0.001	0.28
	6-min walk test distance (m)	−9.53	−21.14, 2.08	0.11	−**0.20**	−**0.34, **−**0.05**	**0.008**	**0.02**	**0.001, 0.04**	**0.04**
Number of Sedentary	Waist circumference (cm)	−0.1	−0.34, 0.14	0.41	−0.05	−0.28, 0.18	0.66	0.006	−0.2, 0.03	0.57
Bouts per Day	Body mass index (kg/m^2^)	0.003	−0.06, 0.06	0.94	−0.02	−0.09, 0.05	0.58	0.001	−0.004, 0.01	0.61
	Systolic blood pressure (mmHg)^b^	**0.87**	**0.29, 1.46**	**0.003**	0.26	−0.30, 0.82	0.376	−**0.07**	−**0.12, **−**0.01**	**0.01**
	MacNew Global	0.008	−0.03, 0.04	0.67	−0.004	−0.05, 0.05	0.89	0.001	−0.002, 0.01	0.37
	HADS-total	0.002	−0.18, 0.19	0.98	**0.13**	**0.01, 0.26**	**0.04**	0.002	−0.02, 0.02	0.82
	Total cholesterol (mmol/L)^c^	**0.04**	**0.002, 0.07**	**0.04**	**0.02**	**0.002, 0.05**	**0.03**	0	−0.004, 0.004	0.93
	High-density lipoprotein (mmol/L)^c^	0.01	−0.002, 0.02	0.14	−0.002	−0.01, 0.004	0.44	4.0E−5	−0.001, 0.001	0.93
	Blood glucose level (mmol/L)^d^	0.02	−0.01, 0.05	0.23	0.02	−0.01, 0.04	0.29	0.002	−0.004, 0.008	0.49
	6-min walk test distance (m)	0.83	−2.30, 3.95	0.60	−2.33	−4.86, 0.20	0.07	0.23	−0.03, 0.49	0.08
Duration of Sedentary	Waist circumference (cm)	−0.17	−0.74, 0.41	0.57	0.06	−0.22, 0.35	0.66	0.01	−0.02, 0.04	0.55
Bouts per Day (min)	Body mass index (kg/m^2^)	−0.03	−0.17, 0.12	0.74	−0.02	−0.1, 0.06	0.67	0.003	−0.01, 0.01	0.54
	Systolic blood pressure (mmHg)^b^	0.6	−1.08, 2.24	0.5	0.27	−0.67, 1.2	0.58	−0.015	−0.10, 0.072	0.73
	MacNew Global	−0.04	−0.13, 0.06	0.43	−0.05	−0.11, 0.003	0.06	0.003	−0.003, 0.01	0.30
	HADS-total	−0.01	−0.53, 0.51	0.97	0.32	−0.001, 0.64	0.051	0.003	−0.02, 0.03	0.84
	Total cholesterol (mmol/L)^c^	0.06	−0.03, 0.14	0.22	0.002	−0.04, 0.04	0.90	−0.002	−0.01, 0.003	0.53
	High-density lipoprotein (mmol/L)^c^	0.01	−0.02, 0.03	0.68	−0.002	−0.02, 0.01	0.83	0	−0.001, 0.001	0.82
	Blood glucose level (mmol/L)^d^	0.03	−0.12, 0.2	0.69	0.02	−0.06, 0.09	0.70	3.8E−5	−0.01, 0.01	0.99
	6-min walk test distance (m)	4.39	−3.82, 12.6	0.3	−1.58	−6.05, 2.9	0.49	−0.07	−0.49, 0.35	0.74
Number of Sedentary	Waist circumference (cm)	−0.1	−0.34, 0.14	0.41	−0.05	−0.28, 0.18	0.66	0.01	−0.02, 0.03	0.58
Breaks per Day	Body mass index (kg/m^2^)	0.003	−0.06, 0.06	0.92	−0.02	−0.09, 0.05	0.57	0.001	−0.004, 0.01	0.63
	Systolic blood pressure (mmHg)^b^	**0.87**	**0.29, 1.44**	**0.003**	0.26	−0.30, 0.82	0.37	−**0.07**	−**0.12, **−**0.01**	**0.01**
	MacNew Global	0.01	−0.03, 0.04	0.65	−0.004	−0.05, 0.04	0.88	0.001	−0.002, 0.01	0.37
	HADS-total	0.004	−0.18, 0.19	0.97	**0.13**	**0.01, 0.26**	**0.04**	0.002	−0.02, 0.02	0.82
	Total cholesterol (mmol/L)^c^	**0.04**	**0.003, 0.07**	**0.03**	**0.02**	**0.002, 0.05**	**0.03**	0	−0.004, 0.004	0.93
	High-density lipoprotein (mmol/L)^c^	0.01	−0.002, 0.02	0.13	−0.002	−0.008, 0.003	0.43	4.5E−5	−0.001, 0.001	0.92
	Blood glucose level (mmol/L)^d^	0.02	−0.01, 0.05	0.22	0.02	−0.01, 0.04	0.29	0.002	−0.004, 0.008	0.49
	6-min walk test distance (m)	0.85	−2.24, 3.93	0.59	−2.32	−4.85, 0.20	0.07	0.23	−0.03, 0.49	0.08

^a^All models adjusted for total counts/day, age, gender, education and employment. Significant results are highlighted in bold. HADS, hospital anxiety and depression scale

^b^Model also adjusted for blood pressure medications

^c^Models also adjusted for cholesterol medications

^d^Models also adjusted for type 2 diabetes

Sensitivity analyses revealed no changes to the magnitude and direction of the findings for MVPA, LIPA and sedentary behaviour, with similar p-values, when models were not adjusted for total accelerometer counts/day (Additional file 2: Tables S1 and S2). Results were also similar for on-protocol and unadjusted models.

## Discussion

Physical inactivity and sedentary behaviour are important risk factors for cardiovascular disease incidence and mortality but their effect on other cardiovascular disease risk factors in cardiac rehabilitation attendees is limited. We found any intensity of physical activity in people who attended cardiac rehabilitation was associated with a decrease in total cholesterol, regardless of time. Higher MVPA was associated with decreased SBP, whereas higher LIPA was associated with decreased measures of adiposity. Higher MVPA was associated with decreased HDL but this change in HDL was negligible over time. How sedentary behaviour is distributed throughout the day may be important in individuals with CHD who attended cardiac rehabilitation as more sedentary behaviour bouts and breaks per day were associated with increased total cholesterol, anxiety and depression. Additionally, an increase in sedentary behaviour bouts and breaks over time was associated with a decrease in systolic blood pressure. These relationships did not vary by age, gender, accelerometer total counts, education and employment status or whether or not participants were taking blood pressure or cholesterol medications or were diagnosed with type 2 diabetes. Further investigation of LIPA and the distribution of sedentary behaviour is indicated, to gain further insight into the relationship between LIPA, sedentary behaviour bouts and breaks and their relationship with cardiovascular risk factors in this population.

As previously reported [21], sedentary behaviour is high (11 h/day) and physical activity is low in this cohort over the 12-month trial period. There was with no change in sedentary behaviour and MVPA during the 6-week cardiac rehabilitation program but both significantly improved over 6-months, with no further improvement at 12-months. LIPA significantly increased during the 6-week cardiac rehabilitation program and this was maintained to 12-months. Only 15% of participants met the public health physical activity guidelines at 6-weeks, with no significant increase in this proportion at 12-months (21%). In contrast to previous studies conducted in cardiac rehabilitation attendees [14, 15], higher amounts of MVPA did not result in decreased BMI and waist circumference, and increased HDL. In fact, in our study adiposity increased and HDL decreased with each minute of MVPA completed. This may be a result of the way MVPA was measured in these studies, with pedometer steps not accounting for intensity of physical activity, or it may be evidence for the ‘active couch potato phenomenon’ [19]. Participants may be completing some MVPA but then spending most of their day sitting, which may also explain a decrease in 6MWTD, with 6MWTD also found to decrease with increases in sedentary behaviour. Interestingly, higher amounts of LIPA resulted in decreases in BMI and waist circumference. These participants may not be achieving MVPA but may be moving more throughout their day. A systematic review has found some evidence that increased LIPA may reduce adiposity and improve lipidaemia but the evidence is limited [30].

Increased MVPA was associated with decreased total cholesterol levels, which is supported by previous cardiac rehabilitation studies [14, 15]. Additionally, increased total cholesterol was associated with increased sedentary behaviour bouts and breaks, however was not associated with increased total sedentary time as found in a similar study [16]. Furthermore, increased LIPA resulted in decreases in total cholesterol, indicating that any level of physical activity is important for cholesterol management. As highlighted above, there is some evidence that increased LIPA may improve lipidaemia, supporting the public health physical activity message that any activity is better than nothing [30]. Higher levels of LIPA may be easier for people with CHD to achieve compared to MVPA and may have some effect on risk factors for recurrent cardiac events in this population. Thus, LIPA should be considered in future CHD and cardiac rehabilitation studies investigating the associations between physical activity and health outcomes.

No previous studies in cardiac rehabilitation reported an association between increased MVPA and decreased systolic blood pressure, although this is well documented in healthy populations and people with hypertension [18]. Pharmacological interventions targeting blood pressure control is one of the cornerstones of treatment for CHD [31], therefore the effect of physical activity beyond medical management may be difficult to determine. We also found an effect of increased sedentary behaviour bouts and breaks on decreasing systolic blood pressure over time. Interrupting sitting time with frequent (every 20–60 min), short bouts (2–3 min) of LIPA has been shown to decrease resting systolic blood pressure in healthy, overweight/obese, type 2 diabetic and stroke populations [32]. Again, breaking up long periods of sitting may be a more achievable strategy than aiming for increased MVPA in people with CHD, at least in the short to moderate-term, with encouragement to increase their physical activity levels and intensity over time.

Anxiety and depression symptoms were found to increase with an increased number of sedentary behaviour bouts and breaks but were not associated with total sedentary time. Bakker et al [13] reported the presence of cardiac anxiety was associated with higher levels of self-reported sedentary behaviour in patients with CHD. This difference in findings may be due to different methods of measurement (self-report vs device), or an increase in sedentary behaviour bouts and breaks may be correlated with increased time in sedentary behaviour and thus acting as a proxy for higher levels of sedentary behaviour [16]. In the same study, sedentary behaviour was measured with a device and cardiac rehabilitation participants were compared to age-matched controls. Patients with CHD demonstrated significantly more prolonged uninterrupted sedentary bouts compared to controls. This association between anxiety, depression and the distribution of sedentary behaviour in cardiac rehabilitation is worthy of further investigation as it may assist with identifying cardiac rehabilitation participants who are at risk of high levels of sedentary behaviour which result in detrimental health outcomes, or in turn, it may assist with managing anxiety and depression by encouraging less sitting time. Either way the pattern of sedentary behaviour may be important for the management of anxiety, depression, total cholesterol and systolic blood pressure in people with CHD.

The investigation of the association of physical activity or sedentary behaviour and risk factors, exercise capacity and health-related quality-of-life in cardiac rehabilitation attendees over time is a strength of this study, although there are several limitations. A high percentage of participants did not have complete accelerometer data, however, we handled missing data by bringing the last value forward and compared participants with and without complete accelerometer data and found no significant differences existed between groups. The participants were also predominantly highly educated males; with normal blood pressure, blood glucose and lipid levels; who had low levels of anxiety and depression and good health-related quality-of-life. This is typical of cardiac rehabilitation populations around the world [33–35] but this does limit generalisability and may explain why no associations were found between physical activity or sedentary behaviour and blood glucose levels and health-related quality-of-life. There is also a possible dilution effect for the accelerometer data due to the averaging of data and the application of a time filter, and this may not accurately reflect the amount of physical activity and sedentary behaviour undertaken by participants. A number of statistical tests were conducted and a Bonferroni correction was applied. The majority of associations did not reach the required threshold, although we have reported the associations significant at *p* < 0.05. These results should be interpreted with caution and require further investigation. Finally, the accelerometer cut-points used in this study may not be appropriate for cardiac rehabilitation participants, and may have inaccurately classified physical activity and sedentary behaviour.

## Conclusions

There is some indication that LIPA improves measures of adiposity and cholesterol, while breaking up sedentary behaviour may decrease SBP over time in cardiac rehabilitation attendees, decreasing their risk of recurrent cardiac events. Further investigation of the role of LIPA and patterns of SB (bouts and breaks) on risk factors for cardiovascular disease in people with heart disease is needed, particularly when current levels of MVPA completed in this population is low. From a clinical perspective, this data may indicate that health professionals should be encouraging cardiac rehabilitation attendees to complete any level of activity to receive the health benefits, alongside interrupting long periods of sitting, which is currently not included in cardiac rehabilitation guidelines.

## Supplementary Information

**Additional file 1**. STROBE checklist.

**Additional file 2**. Sensitivity analyses (Table S1 and S2).

## Data Availability

The datasets used and analysed during the current study are available from the corresponding author on reasonable request.

## Abbreviations

CHD: Coronary heart disease
MVPA: Moderate-to-vigorous physical activity
BMI: Body mass index
HDL: High density lipoprotein
6MWTD: 6-Minute walk test distance
LIPA: Light-intensity physical activity
cpm: Counts per minute
MacNew: MacNew Heart Disease Health-related Quality of Life Questionnaire
HADS: Hospital Anxiety and Depression Scale
GEE: Generalised Estimating Equations
