# Supplementary material for: Association of device-measured physical activity and sedentary behaviour with cardiovascular risk factors, health-related quality-of-life and exercise capacity over 12-months in cardiac rehabilitation attendees with coronary heart disease
Source: BMC Sports Sci Med Rehabil. 2022 Sep 7;14:169. doi: 10.1186/s13102-022-00562-7 (PMC9454132; doi:10.1186/s13102-022-00562-7)
Supplement: Supplementary file 2 — Additional file 2. Sensitivity analyses (Table S1 and S2). [file 13102_2022_562_MOESM2_ESM.doc]

Additional Table 1. Associations between Physical Activity variables and cardiovascular risk factors, health-related quality-of-life and exercise capacity over 12-months in cardiac rehabilitation attendees without adjustment for total counts/day.

| Independent variable^a^ | Dependent variable | Time | | | Independent variable | | | Time*Independent variable | | |
| --- | --- | --- | --- | --- | --- | --- | --- | --- | --- | --- |
|  |  | β | 95% CI | p-value | β | 95% CI | p-value | β | 95% CI | p-value |
| Moderate-to-Vigorous | Waist circumference (cm) | 0.13 | -0.03, 0.3 | 0.11 | **0.05** | **0, 0.1** | **0.03** | **-0.01** | **-0.01, 0** | **0.03** |
| Physical Activity (min/day) | Body mass index (kg/m^2^) | **0.05** | **0.01, 0.10** | **0.03** | **0.01** | **0, 0.02** | **0.01** | -0.001 | 0, -2E-5 | 0.05 |
|  | Systolic blood pressure (mmHg)^b^ | -0.1 | -0.5, 0.47 | 0.96 | **-0.11** | **-0.2, -0.03** | **0.01** | 0.001 | -0.01, 0.01 | 0.87 |
|  | MacNew Global | **0.02** | **0.01, 0.04** | **0.02** | 0.002 | 0, 0.01 | 0.45 | 0.001 | -2E-5, 0 | 0.06 |
|  | HADS-total | -0.03 | -0.15, 0.1 | 0.69 | 0.01 | -0.02, 0.03 | 0.59 | -0.001 | 0, 0 | 0.71 |
|  | Total cholesterol (mmol/L)^c^ | -0.01 | -0.03, 0.01 | 0.39 | **-0.01** | **-0.01, 0** | **0.001** | **0.001** | **0, 0** | **0.01** |
|  | High-density lipoprotein (mmol/L)^c^ | 0 | -0.01, 0.01 | 0.85 | **-0.002** | **0, 0** | **<0.001** | **0** | **6E-5, 0** | **0.01** |
|  | Blood glucose level (mmol/L)^d^ | 0.03 | -0.04, 0.09 | 0.41 | 0.001 | -0.01, 0.01 | 0.8 | 6E-6 | 0,0 | 0.99 |
|  | 6-minute walk test distance (m) | **2.78** | **0.91, 4.6** | **0.004** | **-0.44** | **-0.86, -0.02** | **0.04** | 0.7 | -0.01, 0.14 | 0.09 |
|  |  |  |  |  |  |  |  |  |  |  |
| Light-intensity | Waist circumference (cm) | 0.19 | -0.22, 0.6 | 0.37 | -0.02 | 0.05, 0.01 | 0.22 | -0.003 | 0,0 | 0.25 |
| Physical Activity (min/day) | Body mass index (kg/m^2^) | 0.08 | -0.07, 0.23 | 0.31 | -0.01 | -0.02, 0.01 | 0.28 | -0.001 | 0,0 | 0.44 |
|  | Systolic blood pressure (mmHg)^b^ | -0.3 | -1.1, 0.52 | 0.47 | -0.01 | -0.08, 0.06 | 0.73 | 0.01 | 0, 0.02 | 0.17 |
|  | MacNew Global | 0.04 | 0, 0.09 | 0.06 | 0.004 | 0, 0.01 | 0.1 | 0 | 0, 0 | 0.47 |
|  | HADS-total | -0.05 | -0.33, 0.23 | 0.75 | 0.001 | -0.02, 0.03 | 0.93 | -5E-5 | 0,0 | 0.97 |
|  | Total cholesterol (mmol/L)^c^ | **0.07** | **0.01, 0.14** | **0.03** | -0.01 | -0.01, 2E-5 | 0.05 | -0.001 | 0, 6E-5 | 0.08 |
|  | High-density lipoprotein (mmol/L)^c^ | 0.01 | 0, 0.02 | 0.07 | 9E-5 | 0, 0 | 0.88 | -2E-5 | 0, 0 | 0.74 |
|  | Blood glucose level (mmol/L)^d^ | 0.09 | -0.01, 0.2 | 0.09 | 0.001 | 0, 0.01 | 0.78 | -0.001 | 0, 0 | 0.17 |
|  | 6-minute walk test distance (m) | **7.49** | **3.4, 11.57** | **<0.001** | **0.59** | **0.12, 1.07** | **0.02** | -0.04 | -0.08, 0.01 | 0.1 |

^a^ All models adjusted for age, gender, education and employment. Significant results are highlighted in bold. HADS, hospital anxiety and depression scale; ^b^Model also adjusted for blood pressure medications; ^c^Models also adjusted for cholesterol medications; ^d^Models also adjusted for type 2 diabetes.

Additional Table 2. Associations between Sedentary Behaviour variables and cardiovascular risk factors, health-related quality-of-life and exercise capacity over 12-months in cardiac rehabilitation attendees without adjustment for total counts/day.

| Independent variable^a^ | Dependent variable | Time | | | Independent variable | | | Time*Independent variable | | |
| --- | --- | --- | --- | --- | --- | --- | --- | --- | --- | --- |
|  |  | β | 95% CI | p-value | β | 95% CI | p-value | β | 95% CI | p-value |
| Sedentary Behaviour | Waist circumference (cm) | -0.38 | -1.29, 0.53 | 0.41 | -0.01 | -0.02, 0.01 | 0.35 | 0 | 0, 0 | 0.51 |
| (min/day) | Body mass index (kg/m^2^) | 0 | -0.24, 0.25 | 0.98 | 0 | 0, 0 | 0.70 | 5E-6 | 0, 0 | 0.98 |
|  | Systolic blood pressure (mmHg)^b^ | 1.3 | -1.66, 4.25 | 0.39 | -0.01 | -0.04, 0.02 | 0.63 | -0.002 | -0.01, 0 | 0.43 |
|  | MacNew Global | 0.04 | -0.1, 0.17 | 0.60 | 0 | 0, 0 | 0.80 | -7E-6 | 0,0 | 0.95 |
|  | HADS-total | -0.53 | -1.27, 0.21 | 0.16 | 0 | -0.01, 0.01 | 0.96 | 0.001 | 0, 0 | 0.16 |
|  | Total cholesterol (mmol/L)^c^ | -0.08 | -0.23, 0.07 | 0.31 | 0 | 0, 0 | 0.71 | 0 | -8E-5, 0 | 0.21 |
|  | High-density lipoprotein (mmol/L)^c^ | 0 | -0.04, 0.04 | 0.92 | 0 | 0, 0 | 0.16 | 1E-5 | -4E-5, 7E-5 | 0.63 |
|  | Blood glucose level (mmol/L)^d^ | -0.3 | -0.8, 0.2 | 0.24 | 0 | 0, 0 | 0.92 | 0.001 | 0, 0 | 0.21 |
|  | 6-minute walk test distance (m) | -6.44 | -17.13, 4.25 | 0.24 | **-0.24** | **-0.37, -0.11** | **<0.001** | 0.02 | 0, 0.03 | 0.07 |
|  |  |  |  |  |  |  |  |  |  |  |
| Number of Sedentary | Waist circumference (cm) | -0.11 | -0.35, 0.12 | 0.36 | -0.02 | -0.24, 0.21 | 0.87 | 0.01 | -0.02, 0.03 | 0.59 |
| Bouts per Day | Body mass index (kg/m^2^) | 0 | -0.06, 0.06 | 0.96 | 0 | -0.07, 0.06 | 0.85 | 0 | 0, 0.01 | 0.63 |
|  | Systolic blood pressure (mmHg)^b^ | **0.87** | **0.29, 1.45** | **0.003** | 0.33 | -0.15, 0.79 | 0.18 | **-0.07** | **-0.12, -0.01** | **0.01** |
|  | MacNew Global | 0.01 | -0.03, 0.05 | 0.60 | -0.02 | -0.06, 0.02 | 0.33 | 0.001 | 0, 0.01 | 0.39 |
|  | HADS-total | 0 | -0.19, 0.19 | 1.0 | **0.13** | **0.01, 0.26** | **0.03** | 0.002 | -0.02, 0.02 | 0.81 |
|  | Total cholesterol (mmol/L)^c^ | **0.04** | **0, 0.07** | **0.03** | **0.04** | **0.01, 0.07** | **0.02** | 0 | 0, 0 | 0.92 |
|  | High-density lipoprotein (mmol/L)^c^ | 0.01 | 0, 0.02 | 0.14 | 0 | -0.01, 0 | 0.15 | 8E-5 | 0, 0 | 0.85 |
|  | Blood glucose level (mmol/L)^d^ | 0.01 | -0.02, 0.04 | 0.37 | 0.03 | -0.01, 0.06 | 0.11 | 0.003 | 0, 0.01 | 0.32 |
|  | 6-minute walk test distance (m) | 1.15 | -2.01, 4.3 | 0.48 | **-3.89** | **-6.32, -1.46** | **0.002** | 0.23 | -0.04, 0.51 | 0.09 |
|  |  |  |  |  |  |  |  |  |  |  |
| Duration of Sedentary | Waist circumference (cm) | -0.10 | -0.31, 0.11 | 0.33 | 0 | -0.1, 0.01 | 0.94 | 0 | 0, 0 | 0.51 |
| Bouts per Day (min) | Body mass index (kg/m^2^) | 0 | -0.05, 0.05 | 0.96 | 0 | 0, 0 | 0.84 | 7E-5 | 0, 0 | 0.56 |
|  | Systolic blood pressure (mmHg)^b^ | **0.77** | **0.25, 1.29** | **0.003** | 0.02 | -0.01, 0.04 | 0.13 | **-0.003** | **-0.01, 0** | **0.02** |
|  | MacNew Global | 0.01 | -0.02, 0.04 | 0.63 | 0 | 0, 0 | 0.17 | 7E-5 | -8E-5, 0 | 0.39 |
|  | HADS-total | 0.01 | -0.16, 0.18 | 0.89 | **0.01** | **0, 0.01** | **0.03** | 0 | 0, 0 | 0.72 |
|  | Total cholesterol (mmol/L)^c^ | **0.04** | **0.1, 0.07** | **0.01** | **0.002** | **0, 0** | **0.02** | -3E-5 | 0, 0 | 0.70 |
|  | High-density lipoprotein (mmol/L)^c^ | 0.01 | 0, 0.02 | 0.08 | 0 | 0, 0 | 0.24 | 2E-6 | -4E-5, 4E-5 | 0.93 |
|  | Blood glucose level (mmol/L)^d^ | 0.03 | -0.01, 0.06 | 0.15 | 0.001 | 0, 0 | 0.17 | 9E-5 | 0,0 | 0.39 |
|  | 6-minute walk test distance (m) | 1.93 | -1.0, 4.86 | 0.20 | **-0.17** | **-0.27, -0.07** | **0.001** | 0.01 | -0.01, 0.02 | 0.23 |
|  |  |  |  |  |  |  |  |  |  |  |
| Number of Sedentary | Waist circumference (cm) | -0.11 | -0.34, 0.12 | 0.36 | -0.02 | -0.24, 0.21 | 0.87 | 0.006 | -0.02, 0.03 | 0.59 |
| Breaks per Day | Body mass index (kg/m^2^) | -0.001 | -0.06, 0.06 | 0.97 | -0.006 | -0.07, 0.06 | 0.84 | 0.001 | 0, 0.01 | 0.65 |
|  | Systolic blood pressure (mmHg)^b^ | **0.86** | **0.29, 1.44** | **0.003** | 0.32 | -0.15, 0.79 | 0.18 | **-0.07** | **-0.12, -0.01** | **0.01** |
|  | MacNew Global | 0.01 | -0.03, 0.05 | 0.59 | -0.02 | -0.06, 0.02 | 0.33 | 0.001 | 0, 0.01 | 0.39 |
|  | HADS-total | 0.001 | -0.18, 0.18 | 0.99 | **0.13** | **0.01, 0.26** | **0.03** | 0.002 | -0.02, 0.02 | 0.82 |
|  | Total cholesterol (mmol/L)^c^ | **0.04** | **0, 0.07** | **0.03** | **0.04** | **0.01, 0.07** | **0.02** | 0 | 0,0 | 0.92 |
|  | High-density lipoprotein (mmol/L)^c^ | 0.01 | 0, 0.02 | 0.14 | -0.004 | -0.01, 0.002 | 0.15 | 9E-5 | 0, 0 | 0.84 |
|  | Blood glucose level (mmol/L)^d^ | 0.01 | -0.02, 0.04 | 0.35 | 0.03 | -0.01, 0.06 | 0.11 | 0.003 | 0, 0.01 | 0.31 |
|  | 6-minute walk test distance (m) | 1.16 | -1.95, 4.28 | 0.46 | **-3.89** | **-6.3, -1.46** | **0.002** | 0.24 | -0.03, 0.51 | 0.09 |

^a^ All models adjusted for age, gender, education and employment. Significant results are highlighted in bold. HADS, hospital anxiety and depression scale; ^b^Model also adjusted for blood pressure medications; ^c^Models also adjusted for cholesterol medications; ^d^Models also adjusted for type 2 diabetes.
